# The Self-Paced Graz Brain-Computer Interface: Methods and Applications

**DOI:** 10.1155/2007/79826

**Published:** 2007-08-30

**Authors:** Reinhold Scherer, Alois Schloegl, Felix Lee, Horst Bischof, Janez Janša, Gert Pfurtscheller

**Affiliations:** ^1^Laboratory of Brain-Computer Interfaces, Institute for Knowledge Discovery, Graz University of Technology, Krenngasse 37, 8010 Graz, Austria; ^2^NeuroCenter Styria, Krenngasse 37/I, 8010 Graz , Austria; ^3^Intelligent Data Analysis Group, Fraunhofer-Institut für Rechnerarchitektur und Softwaretechnik, FIRST, Kekulestrasse 7, 12489 Berlin, Germany; ^4^Institute for Computer Graphics and Vision, Graz University of Technology, Inffeldgasse 16, 8010 Graz, Austria; ^5^Aksioma - Institute for Contemporary Art, Neubergerjeva 25, 1000 Ljubljana, Slovenia

## Abstract

We present the self-paced 3-class Graz brain-computer interface (BCI) which is based on the detection of sensorimotor electroencephalogram (EEG) rhythms induced by motor imagery. Self-paced operation means that the BCI is able to determine whether the ongoing brain activity is intended as control signal (intentional control) or not (non-control state). The presented system is able to automatically reduce electrooculogram (EOG) artifacts, to detect electromyographic (EMG) activity, and uses only three bipolar EEG channels. Two applications are presented: the freeSpace virtual environment (VE) and the Brainloop interface. The freeSpace is a computer-game-like application where subjects have to navigate through the environment and collect coins by autonomously selecting navigation commands. Three subjects participated in these feedback experiments and each learned to navigate through the VE and collect coins. Two out of the three succeeded in collecting all three coins. The Brainloop interface provides an interface between the Graz-BCI and Google Earth.

## 1. INTRODUCTION

A brain-computer interface (BCI) transforms electrophysiological or metabolic brain activity into control signals for applications and devices (e.g., spelling system or neuroprosthesis). Instead of muscle activity, a specific type of mental activity is used to operate such a system. For a review on BCI technologies see [1–4].

After years of basic research, modern BCIs have been moving out of the laboratory and are under evaluation in hospitals and at patients' homes (e.g., [5–11]). However, BCIs have to meet several technical requirements before they are practical alternatives to motor controlled communication devices. The most important requirements are high information transfer rates, ease-of-use, robustness, on-demand operability, and safety [12]. In summary, for the end-user, BCI systems have to carry information as quickly and accurately as needed for individual applications, have to work in most environments, and should be available without the assistance of other people (self-initiation). To fulfill these issues, the Graz group focused on the development of small and robust systems which are operated by using one or two bipolar electroencephalogram (EEG) channels only [13]. Motor imagery (MI), that is, the imagination of movements, is used as the experimental strategy.

In this work, we aim at two important issues for practical BCI systems. The first is detection of electromyographic (EMG) and reduction of electrooculographic (EOG) artifacts and the second is the self-paced operation mode. Artifacts are undesirable signals that can interfere and may change the characteristics of the brain signal used to control the BCI [14]. Especially in early training sessions, EMG artifacts are present in BCI training [15]. It is therefore crucial to ensure that (i) brain activity and not muscle activity is used as source of control and that (ii) artifacts are not producing undesired BCI output. Self-paced BCIs are able to discriminate between intentionally generated (intentional control, IC) and ongoing (non-control, NC) brain activity [16]. This means that the system is able to determine whether the ongoing brain pattern is intended as control signals (IC) or not (NC). In this mode the user has control over timing and speed of communication.

In addition to the above stated methods, two applications, designed for self-paced operation, are presented. The first is a computer game like virtual environment (VE) that users navigate through and collect points, and the second is a user-interface which allows operating the Google-Earth (Google, Mountain View, CA, USA) application.

## 2. METHODS

### 2.1. Electromyography (EMG) artifact detection

The results of [17] showed that muscle and movement artifacts can be well detected by using the principle of inverse filtering. The inverse filtering method aims to estimate an autoregressive (AR) filter model (see (1)) of the EEG activity. The output *y_t_* of the AR model is the weighted sum of the number of model order *p* last sample values *y*_*t−i*_ and the model parameters *a*_*i*_ with *i* = 1…*p*. *v*_*t*_ is a zero-mean-Gaussian-noise with variance *σ* *_v_* ^2^. Applying the filter model inversely (using the inverted transfer functions) to the measured EEG yields a residual (i.e., prediction error) which is, usually, much smaller than the measured EEG. In effect, all EEG frequencies are suppressed. If some transient or other high-frequency artifacts (like EMG artifacts) are recorded at the EEG channels, the average prediction error will increase. This increase can be detected by computing the time-varying root-mean-square (RMS) of the residual and comparing it with a predefined threshold value. Once the AR parameters *a_i_* are identified from an artifact free EEG segment, these parameters can be applied inversely to estimate the prediction error *x_t_* from the observed EEG *y_t_*,

(1) $$y_{t}=\overset{p}{\underset{i=1}{\sum }}a_{i}\cdot y_{t-i}+\nu_{t}\;\text{with}\;\nu_{t}=N(0,\sigma_{\nu }^{2}).$$

For on-line experiments, the detection threshold of five times RMS from artifact-free EEG was used. Each time the inversely filtered process exceeded this threshold, the occurrence of an EMG artifact in form of a yellow warning marker, positioned in the lower-left part of the screen, was reported back to the user. After any occurrence, users were instructed to relax until the warning disappeared. The warning was disabled once the threshold was not exceeded for a 1-second period.

**Figure 1:**
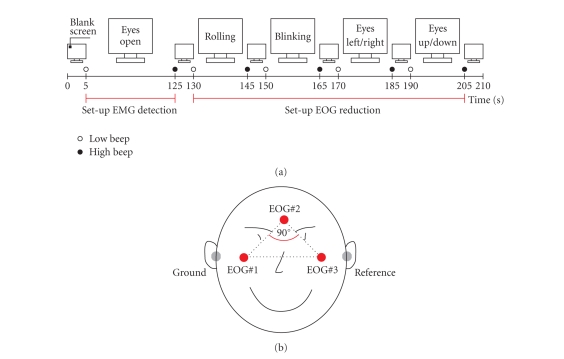
(a) Protocol used for the collection of EEG and EOG samples to set up the EMG detection and EOG reduction. The recording was divided into several segments, each separated by a 5-s resting period. Instructions were presented on a computer screen. At the beginning and end of each task low -and high-warning tones were presented, respectively. (b) Positions of EOG electrodes (reference left mastoid, ground right mastoid). The three EOG electrodes are placed above the nasion, and below the outer canthi of the eyes, generating in a right-angled triangle. The legs of the triangle form two spatially orthogonal components (modified from [18]).

At the beginning of each BCI session, a 2-minute segment of artifact free EEG was recorded in order to estimate the AR-parameters *a_i_* (model order *p* = 10) by using the Burg method. See Figure 1(a) for details on the protocol used to collect the artifact free EEG. Subjects were instructed to sit relaxed and not move.

### 2.2 Automatic reduction of electrooculography (EOG) artifacts

Electrooculogram (EOG) artifacts are potential shifts on the body surface resulting from retinal dipole movements or blinking of the eyes. Since generally both eyes are in the same line of sight, one single dipole consisting of three spatial components (horizontal, vertical, and radial) should be sufficient to model the bioelectrical eye activity [18]. Assuming that (i) for each channel the recorded EEG *Y_t_* is a linear superposition of the real EEG signal *S_t_* and the three spatial EOG components ( *N*_*t*,horizontal_, *N*_*t*,vertical_, and *N*_*t*,radial_) weighted by some coefficient *b* (2) and that (ii) EEG S and EOG **N** are independent, the weighting coefficient **b** can be estimated according to (3) (matrix notation) by computing the autocorrelation matrix **C**_*N,N*_ of the EOG and the cross-correlation **C**_*N,Y*_ between EEG **Y** and EOG **N**. Equation (4) describes how the “EOG-corrected” EEG is computed.

(2) $$\begin{array}{ll} Y_{\text{channel},t} & =S_{\text{channel},t}+[N_{\text{horizontal},t},N_{\text{vertical},t},N_{\text{radial},t}]\\ & \;\cdot \left[\begin{array}{c} b_{\text{horizontal,channel}}\\ b_{\text{vertical,channel}}\\ b_{\text{radial,channel}}, \end{array}\right] \end{array}$$

(3) $$\text{Y}=\text{S}+\text{N}\cdot \text{b}\Rightarrow \text{b}=({\text{N}}^{T}\text{N}{)}^{-1}{\text{N}}^{T}\text{Y}={\text{C}}_{N,N}^{-1}{\text{C}}_{N,Y},$$

(4) S=Y−N⋅b.

To limit the total number of channels, the EOG was measured by using three monopolar electrodes, from which two bipolar EOG channels, covering the horizontal and the vertical EOG activity, were derived (Figure 1(b)).

In order to compute the weighting coefficient **b**, at the beginning of each BCI session, a 1-minute segment of EEG and EOG with eye movement artifacts was recorded. Subjects were instructed to repetitively perform eye blinks, clockwise and counter-clockwise rolling of the eyes and perform horizontal and vertical eye movements. The eyes should circumscribe the whole field of view without moving the head. Figure 1(a) summarizes the recording protocol used. A more detailed description as well as the evaluation of the EOG correction method can be found in [18].

### 2.3. Self-paced BCI operation

Essential for the development of self-paced motor imagery (MI)-based BCIs is to train (i) the user to reliably induce distinctive brain patterns and to train (ii) the BCI to detect those patterns in the ongoing EEG. In this work, prior to participate in self-paced experiments, subjects learned to generate three different MI patterns by performing cue-based feedback training. The resulting classifier is named CFR_MI_ . Once a reliable classification performance was achieved, a second classifier (CFR_IC_ )was trained to discriminate between characteristic EEG changes induced by MI and the ongoing EEG. Self-paced control was obtained by combining both classifiers. Each time MI-related brain activity was detected by CFR_IC_ the type of motor imagery task was determined by CFR_MI_. If no MI-related activity was detected from CFR_IC_, the output was “0” or “NC.”

**Figure 2:**
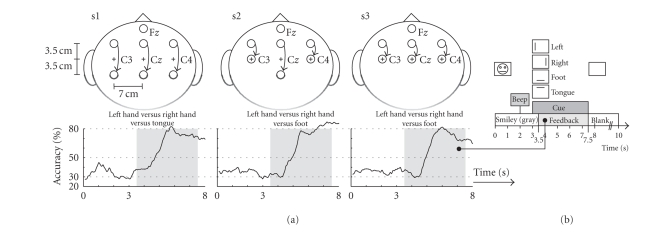
(a) Electrode positions used for self-paced feedback experiments. Fz served as ground. The curves show the average classification accuracy (40 trials/class) of the specified motor imagery tasks. (b) Timing of the cue-based training paradigm. The task was to move a smiley-shaped cursor into the direction indicated by the cue.

Three healthy subjects (2 males, 1 female, right handed) participated in self-paced experiments. Subject specific electrode positions (according to the international 10–20 system), motor imagery tasks and the on-line classification accuracies of CFR_MI_ after about 4 hours of cue-based 3-class feedback training are summarized in Figure 2(a). Three bipolar EEG channels (named C3, C_*z*_, and C4) and three monopolar EOG channels (Figure1(a) ) were recorded from Ag/AgCl electrodes, analog filtered between 0.5 and 100 Hz and sampled at a rate of 250 Hz. Figure 2(b) shows the timing of the cue-based paradigm. Classifier CFR_MI_ was realized by combining 3 pairwise trained Fisher's linear discriminant analysis (LDA) functions with a majority vote. A maximum of six band power (BP) features were extracted from the EEG by band pass filtering the signal (5th-order Butterworth), squaring and applying a 1-second moving average filter. From the averaged value the logarithm was computed (BP_log_).

CFR_IC_ consisted of one single LDA. To identify the most discriminative BP_log_ the distinction sensitive learning vector quantization (DSLVQ [19, 20]) method was used. DSLVQ is an extended learning vector quantizer (LVQ) which employs a weighted distance function for dynamical scaling and feature selection [20]. The major advantage of DSLVQ is that it does not require expertise, nor any a priori knowledge or assumption about the distribution of the data. To obtain reliable values for the discriminative power of each BP_log_ the DSLVQ method was repeated 100 times. For each run of the DSLVQ classification, 50% of the features were randomly selected and used for the training and the remaining 50% were kept to test the classifier. The classifier was fine-tuned with DSLVQ type C training (10000 iterations). The learning rate *α*_*t*_ decreased during this training from an initial value of *α*_*t*_ = 0.05 to *α*_*t*_ = 0. The DSLVQ relevance values were updated with the learning rate *λ*_*t*_ = *α*_*t*_/10.

DSLVQ was applied to the last session of the cue-based feedback training data (4 runs with 30 trials each; 10 per class). From each trial at two time points *t*_1_ and *t*_2_ = *t*_1_ = 1.0 second around the best on-line classification accuracy during motor imagery, ninety-three BP_log_ features were extracted; thirty-one for each bipolar EEG channel between 6–36 Hz with a bandwidth of 2 Hz (1 Hz overlap). Motor imagery tasks were pooled together and labeled as class IC (intentional-control). For class NC (noncontrol), in contrast, BP_log_ were extracted at time of cue onset ( *t* = 3.0 seconds, see Figure 2(b)). This time was selected to prevent the classifier to detect “unspecific” MI preactivations, resulting from the “beep” presented 1 second before the cue. Additionally from the 2-minute segment of artifact free EEG, used to set up EMG detection (Figure 1(b)), in step sizes of 1-second BP_log_ features were extracted. The 6 most discriminative BP_log_ were selected and used to set up CFR_IC_. To increase the robustness of CFR_IC_, an additional threshold TH_IC_ was introduced which had to be exceeded (dropping below) for a subject-specific transition time *t* ^T^ before a switch between NC and IC (IC and NC) occurred. Increasing or decreasing the value of the threshold was synonymous with shifting the optimal LDA decision border. In doing so, at least to some extent, nonstationary changes of the EEG (from session to session) could be adapted.

### 2.4. Navigating the freespace virtual environment

**Figure 3:**
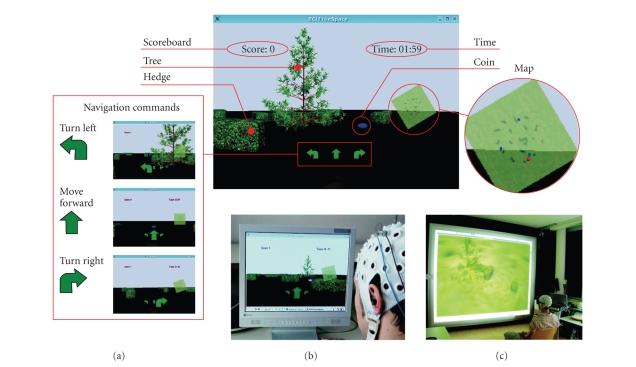
(a) The freeSpace virtual environment. The screenshot shows the tree, some hedges, and a coin to collect. In the lower mid part of the screen, the navigation commands are shown. The number of collected coins and the elapsed time are presented in the upper left and right sides, respectively. For orientation, a map showing the current position (red dot) was presented. (b) Presentation of the VE on a conventional computer screen. (c) Stereoscopic visualization of the VE on a projection wall (perspective point of view).

The “freeSpace” virtual environment (VE) was developed as a platform-independent application based on the Qt application framework (Trolltech, Oslo, Norway) and intended as a test platform for self-paced BCIs. The virtual park consists of a flat meadow, several hedges, and a tree positioned in the middle (Figure 3(a)). Like in computer games, coins were positioned on fixed locations inside the park and users had the task of navigating through the virtual world and collecting them. The coins were automatically picked by contact; hedges or the tree had to be bypassed (collision detection). Four commands were implemented. These are “turn left,” “turn right,” “move forward,” and “no operation.” The user datagram protocol (UDP) was used to realize the communication between BCI and VE. For easier orientation, a map showing the current position was presented (Figure 3(a)). The VE was presented in first-person-view perspective on a conventional computer screen (Figure 3(b)). However, given that proper hardware is available, also a stereoscopic 3D representation is possible (Figure 3(c)). In order to provide feedback on received navigation commands and upcoming state switches as fast as possible, during the transition time *t* ^T^ the command arrows were steadily growing (NC to IC) or shrinking (IC to NC), before performing the requested action.

By autonomously selecting the navigation commands, subjects had the task of picking up the three coins within a three-minute time limit. For each run the starting position inside the VE was altered. No instructions on how to reach the coins were given to the subjects. Two sessions with 3 feedback training runs were recorded. Each session started with free-training lasting about 20 minutes. At the beginning of this period the subject-specific threshold TH_IC_ and transition time *t* ^T^ (maximum value 1 second) were identified empirically according to the statements of the subjects and fixed for the rest of the session. At the end of each session subjects were interviewed on the subjective-experienced classification performance. The BCI and the VE were running on different computers.

For more details on user training, self-paced BCI sytem set-up and evaluation of the freeSpace VE see [21].

### 2.5. Operating Google Earth-Brainloop

**Figure 4:**
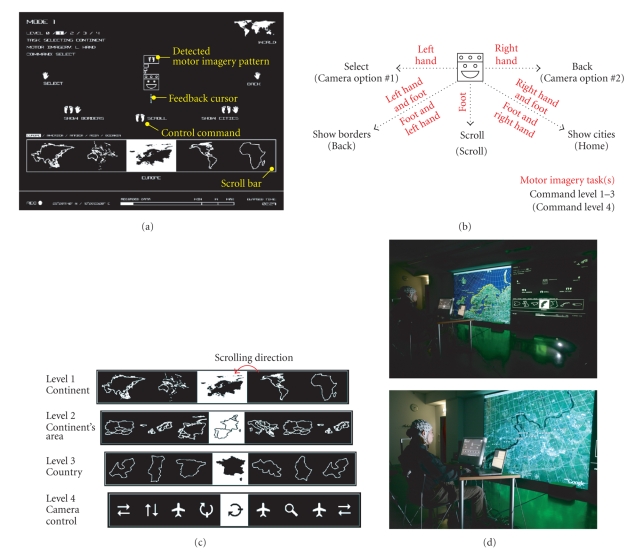
(a) Screenshot of the “Brainloop” interface. The upper part of the screen was used to select the command. The available options were presented in a scroll bar in the lower part of the screen. (b) Available commands for operating Google Earth and used motor imagery tasks. (c) The four levels of selections. (d). Photographs of the “Brainloop” performance.

The Brainloop interface for Google Earth was implemented in Java (Sun Microsystems Inc., Santa Clara, CA, USA). The communication with the BCI was realized by means of the UDP protocol; the communication with Google Earth by using the TCP/IP protocol. A multilevel selection procedure was created to access the whole functional range of Google Earth. Figure 4(a) shows a screen shot of the interface. The user was represented by an icon positioned in the center of the display. The commands at the user's disposal were placed around this icon and could be selected by moving the feedback cursor into the desired direction. The three main commands “scroll,” “select” and “back” were selected by moving the cursor to the left, right, or down, respectively. After each command Google Earth's virtual camera moved to the corresponding position. By combining the cursor movement down with left or right, the commands “show borders” and “show cities” were activated (Figure 4(b)). During the transition time *t* ^T^ the feedback cursor was moving towards the desired control command (NC to IC) or back to the user icon presented in the middle of the screen (IC to NC). Once the feedback cursor was close to the command, this was highlighted and accepted. Figure 4(c) summarizes the four hierarchically arranged selection levels. Levels 1 to 3 were needed to select the continent, the continental area and the country. The scroll bar at level 4 contained commands for the virtual camera (“scan,” “move,” “pan,” “tilt,” and “zoom”). For this level also the assignment of the commands was changed (see Figure 4(b)). Every selection was made by scrolling through the available options and picking the highlighted one. While the “scroll” command was selected, the options were scrolling at a speed of approximately 2 items/s from the right to the left. For more details on the interface please see [22].

Subject s2 took part in self-paced on-line experiments. Figure 4(b) summarizes the MI tasks used to operate the feedback cursor. After three consecutive days of training (about 2.5 hours/day) on December 14, 2006, a media performance lasting about 40 minutes was presented to the audience. Figure 4(d) shows pictures taken during the Brainloop media performance. Because it is very difficult to compute self-paced BCI performance measures, after the media performance the subject self-reported on the BCI classification accuracy.

## 3. RESULTS

**Figure 5:**
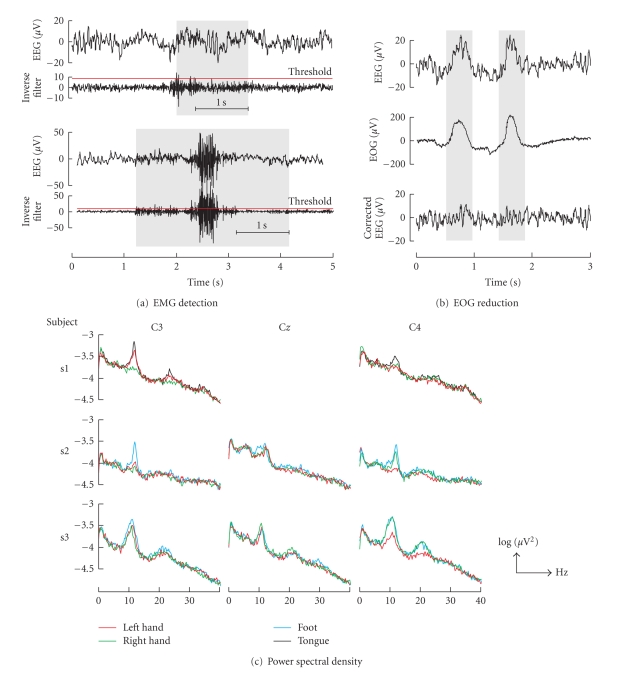
(a) EMG detection example. The EEG and the inverse filter output are shown for minor (upper part) and massive (lower part) EMG artifacts. (b) Example of EOG reduction. The recorded EEG, EOG channel #2, and the corrected EEG are presented in the upper, middle, and lower parts, respectively. (c) Logarithmic power density spectra of cue-based motor imagery trials (40 each) after 4 hours of feedback training.

**Figure 6:**
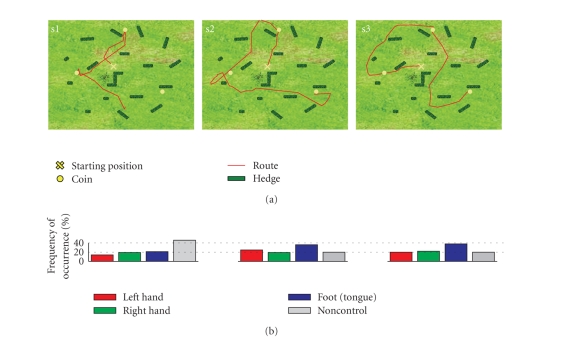
(a) Map of the freeSpace virtual environment showing the best performance (covered distance) for each subject. (b) Frequencies of occurrence of the detected motor-imagery tasks (selected navigation commands).

The percentage of EEG samples classified as EMG artifact during the training procedure was less than 0.9% for each subject. Figure 5(a) shows example EMG detections. The method works well for minor (upper plot) as well as for massive muscle activity (lower plot). The power density spectrum for each channel and motor imagery task is summarized in Figure 6. The power density spectrum was computed by averaging the power spectrum of the forty motor imagery trials for each class recorded during the last cue-based feedback session. Before computing the discrete Fourier transform of the 4-second motor imagery segment (see Figure 2(b)) a hamming window was applied. The spectra show clear peaks in the upper-alpha (10–12 Hz) and upper-beta bands (20–25 Hz).

The EOG reduction method is able to reduce about 80% of EOG artifacts [18]. The example in Figure 5(b) shows clearly that eye blinks were removed from the EEG.

The relevant frequency components for the discrimination between IC and NC identified for each subject by DSLVQ are summarized in Table 1. The therewith trained LDA classifiers achieved classification accuracies (10 × 10 cross-validation) of 77%, 84%, and 78% for subjects s1, s2, and s3, respectively [21].

Each subject successfully navigated through the freeSpace VE and collected coins. Subjects s2 and s3 succeeded in collecting all three items within the 3-minute time limit. Subject s1 was able to collect only 2 of the 3 coins. While s1 and s2 were able to improve their performance (reflected in the covered distance and number of collected items), the results of session two for s3 were poor compared to the first. The best performance for each subject out of the 6 runs recorded on 2 different days is shown in Figure 6. The covered distances (Figure 6(a)), however, show that subjects (depending also on the ability to control the BCI) choose different ways to collect the coins. The corresponding distribution of the BCI classification in Figure 6(b) show that each class occurred during the experiment. Interviews with the subjects confirmed that all 3 motor imagery mental states as well as NC were deliberately used for navigation. The “no operation” command (non-control state) was necessary during non-MI related mental activity, like, for example, orientation or routing, or whenever subjects needed a break.

The Brainloop interface is a very intuitive graphical user interface for Google Earth. The developed selection procedure enables users to quickly pick a country and to manipulate the virtual camera. Although audience was present during the performance, subject s2 succeeded in operating Google Earth. After the performance, the subject stated that most of the time the BCI was correctly detecting the intended motor imagery patterns as well as the non-control state.

## 4. DISCUSSION

The presented BCI is able to automatically reduce EOG artifacts, detect EMG activity, and support the self-paced operation mode. Each of these issues is important for the realization of practical BCI systems. Additionally only three bipolar channels were used which makes the system easy to use and inexpensive compared to a system with more channels.

The proposed EOG reduction and EMG detection methods are simple to implement, computationally not demanding, and can easily be adapted at the beginning of each feedback session. One still open issue, however, is the long-term stability of the methods. Both methods are part of the BioSig open source toolbox [23] and freely available under the GNU General Public License.

Since the proposed EOG reduction method modifies the recorded EEG, an analysis of the influence on the classification accuracy was performed. DSLVQ was applied to the EEG (cue-based training) before and after EOG reduction. The computed DSLVQ feature relevance showed that the same frequency components are relevant before and after applying the EOG reduction method. A statistical analysis of the DSLVQ classification results revealed no significant difference (*P* > 0.05). Since the frequency range of EOG artifacts is maximal at frequencies below 4 Hz (we were looking in the range 8–30 Hz) and prominent over anterior head regions [14], this result was not surprising.

Although both methods have no direct impact on the system classification performance, the robustness of the BCI could be increased. Artifacts did not cause wrong system responses, but were either reduced or detected and reported back. After artifact detection different options are possible. These include a “pause-mode” (or “freeze-mode”) or to “reset” the system to the initial status. In both cases the BCI suspends execution. While in the former case, after a predefined period of artifact-free EEG, the BCI resumes working, in the latter case, the system resets itself. The choice, however,primarily depends on the robustness of the selected signal processing method in the presence of artifacts.

Even though very simple feature extraction and classification methods were used to create a self-paced system, subjects reported they were quite satisfied with the BCI classification performance. An open question is determining the optimum detection threshold TH_IC_ and the transition time *t_T_*. We used an empirical approach and changed the parameters according to the statements of the subjects, which is only a suboptimum solution.

For cue-based systems a variety of different performance measures exist. Since only a few groups investigate asynchronous or self-paced systems [24–26], appropriate benchmark tests and performance measures are not available yet [27].

The “freeSpace“ paradigm was introduced because no instructions, except the overall aim to collect coins, had to be given to the subjects. The paradigm is motivating, entertaining and most important there is an endless number of ways to collect the coins.

The Brainloop interface provides a new way to interact with complex applications like Google Earth. By remapping commands and options the interface can be customized also for other applications. Self-report was selected to characterize BCI performance, since performance can be difficult to measure objectively with asynchronous BCIs. Interesting is that there was no need to adapt the detection threshold TH_IC_ and the transition time *t*_T_. The values fixed during the last freeSpace session were used.

The results of the experiments show that subjects learned to successfully use applications by autonomously switching between different mental states and thereby operating the self-paced Graz-BCI.

## Figures and Tables

**Table 1:** Relevant frequency components in Hz identified by DSLVQ for the discrimination of intentional control (IC) and the non-control state (NC).

Subject	C3	C *z*	C4
s1	12–14, 15–17, 20–22, 25–27	9–11, 21–23	—
s2	12–14, 19–21, 27–29	9–11, 11–13	21–23
s3	8–10, 16–18	8–10	15–17, 24–26
